# EUS–guided abscess drainage in an elderly patient with an abscess in the right liver lobe (with video)

**DOI:** 10.1097/eus.0000000000000059

**Published:** 2024-05-31

**Authors:** Yuki Ikeda, Daichi Watanabe, Ginji Oomori, Shota Yamada, Toshinori Okuda, Shinya Minami

**Affiliations:** Department of Gastroenterology, Oji General Hospital, Tomakomai, Hokkaido, Japan.

Percutaneous drainage is used as first-line drainage for liver abscesses. However, elderly patients sometimes experience delirium, increasing the risk of tubes being pulled out. Recently, EUS–guided abscess drainage (EUS-AD) is considered an alternative method.^[1,2]^ The right liver lobe is distant from the gastrointestinal wall, making EUS-AD of an abscess in the right lobe challenging.^[3]^

**Figure 1:**
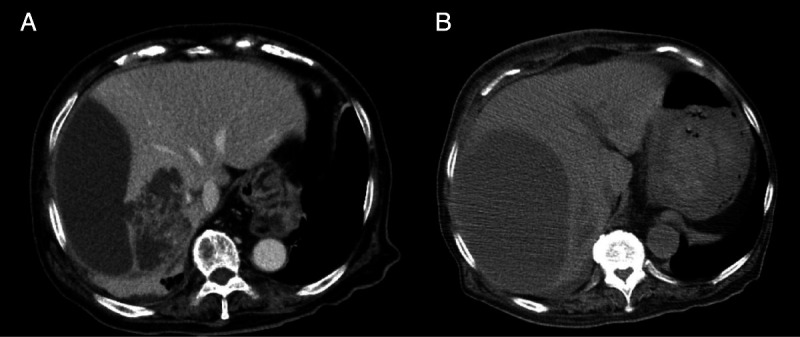
Computed tomography revealed multiple abscesses in the right lobe of the liver (A). The abscess in the right liver lobe was enlarged (B).

**Figure 2:**
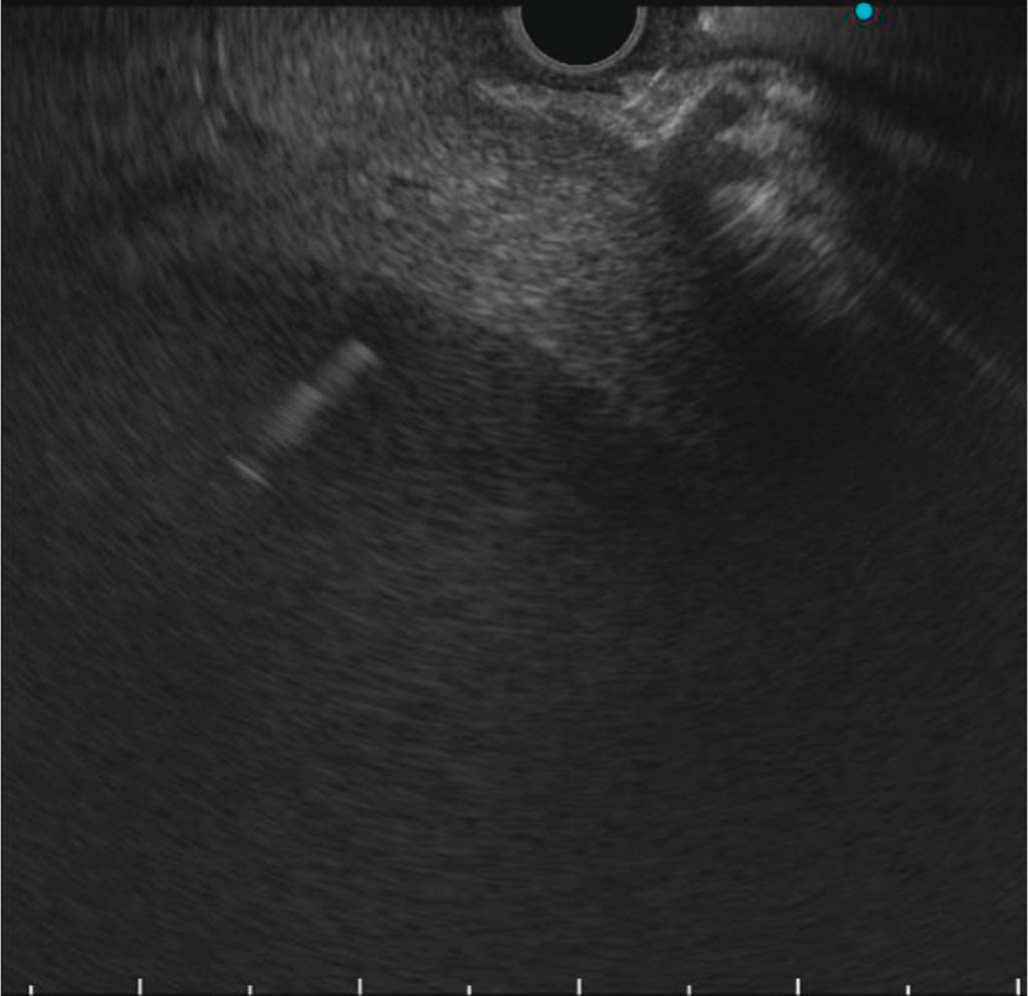
The liver abscess was punctured with a 19-gauge needle from the duodenal wall that followed a 0.025-inch guidewire that was placed within the abscess.

**Figure 3:**
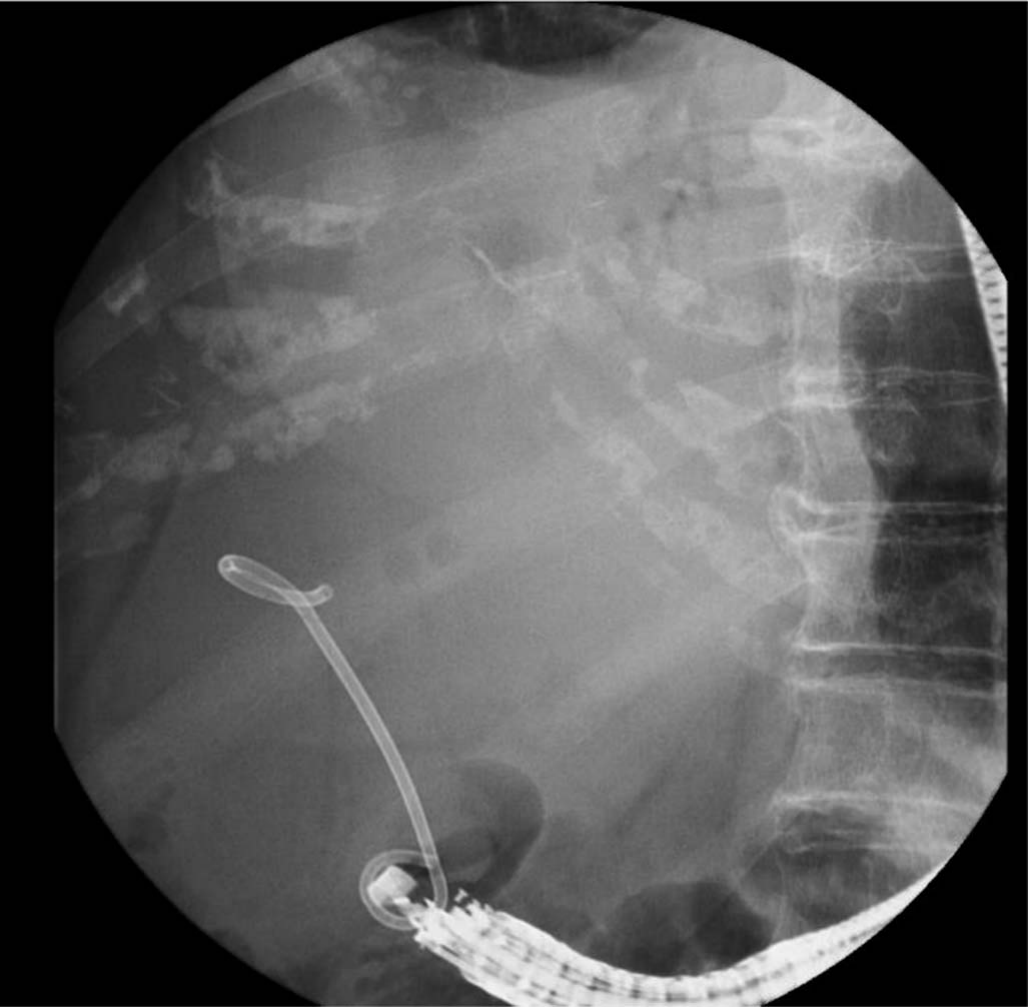
A 7F × 7-cm double-pigtail stent was deployed into the abscess.

**Figure 4:**
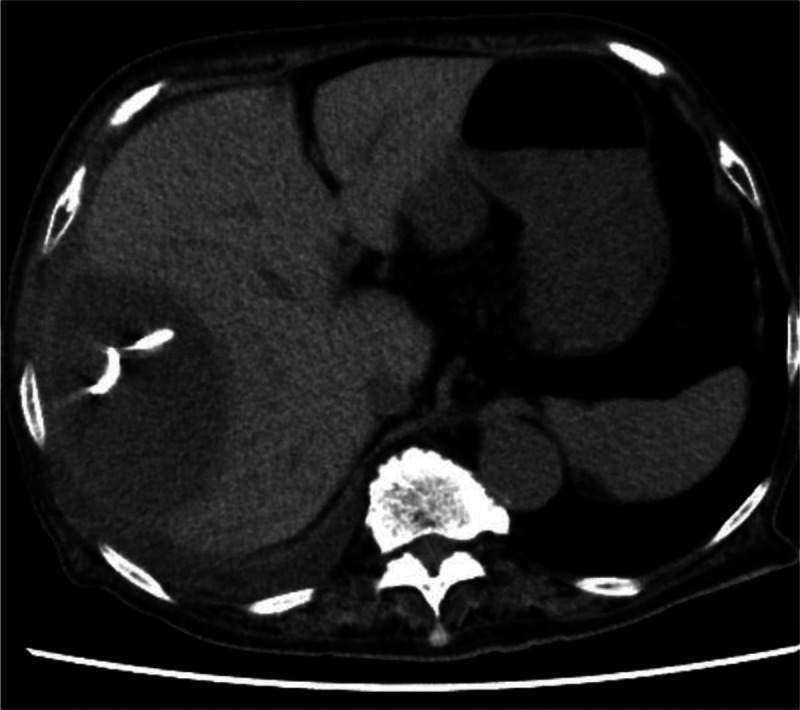
A residual abscess in the right liver lobe.

**Figure 5:**
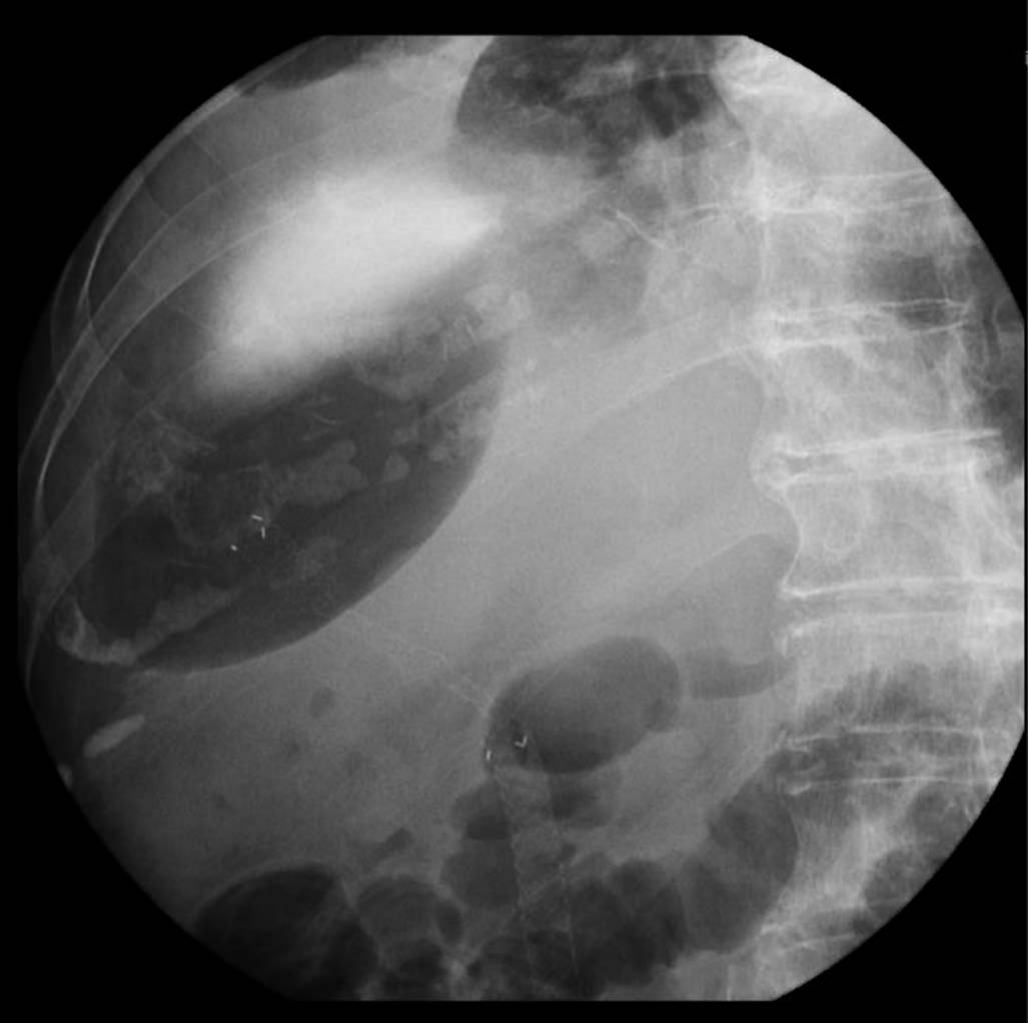
A fully covered self-expandable metallic stent was placed into the liver abscess.

An 89-year-old woman with a high fever, hypotension, and hypoxemia was referred to our hospital. Computed tomography (CT) revealed multiple abscesses in her right liver lobe [Figure 1A]. Antibiotic therapy, oxygen, and a vasopressor were administered. The patient’s blood pressure and respiration gradually improved; however, an enlarged abscess in the right liver lobe was observed [Figure 1B]. The patient suffered from delirium, and therefore tubes risked being pulled out. An EUS-AD was attempted [Video 1] despite the gastrointestinal wall being distant to the right liver lobe. The liver abscess was visualized with convex EUS and punctured with a 19-gauge needle from the duodenal wall. Blood vessels were avoided by using color Doppler ultrasonography. After detecting pus, a 0.025-inch guidewire (VisiGlide 2; Olympus, Tokyo, Japan) was placed within the abscess [Figure 2]. The fistula was dilated using MTW catheter, and then a 0.035-inch guidewire (RevoWave SeekMaster hard; Piolax Medical Devices, Yokohama, Japan) was replaced. A 7F × 7-cm double-pigtail stent (Advanix J; Boston Scientific, Tokyo, Japan) was deployed into the abscess [Figure 3]. CT 2 weeks later revealed a residual right liver lobe abscess [Figure 4]. As endoscopic reintervention (E-RI), a fully covered self-expandable metallic stent (8 mm × 8 cm, HANAROSTENT Benefit; Boston Scientific) was placed into the abscess after removing the double-pigtail stent [Figure 5]. One week after E-RI, the liver abscess had completely resolved, and the stent was removed under fluoroscopic guidance.

In this case, we described EUS-AD in an elderly patient with an abscess in the right liver lobe that was successfully performed. EUS-AD with internal drainage, which has no risk of self-extraction of tubes compared with external drainage, may be one of the treatment options for elderly patients.

EUS–guided abscess drainage of an abscess in the right liver lobe.

Videos are only available at the official website of the journal (http://www.eusjournal.com).
